# The Strontium Ion Reprograms Monocyte Subsets via TRPM2 Channel Regulation to Enhance Osseointegration

**DOI:** 10.34133/bmr.0286

**Published:** 2025-11-24

**Authors:** Congrui Zhao, Antian Xu, Jingyao Gong, Yangbo Xu, Ping Sun, Fuming He

**Affiliations:** ^1^ Stomatology Hospital, School of Stomatology, Zhejiang University School of Medicine, Zhejiang Provincial Clinical Research Center for Oral Diseases, Zhejiang Key Laboratory of Oral Biomedical, Hangzhou 310000, China.; ^2^ Engineering Research Center of Oral Biomaterials and Devices of Zhejiang Province, Hangzhou 310000, China.

## Abstract

Early immune homeostasis at the biomaterial–tissue interface is a critical engineering challenge for osseointegration success. While strontium (Sr)-modified biomaterials exhibit advantages in enhancing osseointegration, the immunomodulatory effects of localized Sr release, particularly on upstream monocytes, remain unelucidated. This study aims to delineate Sr-reprogrammed monocyte subset dynamics and the underlying mechanism. Here, we engineered Sr-doped sandblasted, large-grit, and acid-etched (Sr-SLA) titanium implants. Sr-SLA implants ameliorated the early inflammatory microenvironment and promoted osseointegration. To decipher the Sr-modulated immune microenvironment, we employed single-cell RNA sequencing, which revealed that monocytes constituted the largest proportion of cells surrounding implants, with subset distribution correlating with osteogenic efficiency. Notably, Sr-SLA implants suppressed the activation of pro-inflammatory classical monocytes (Ly6C^hi^), with high transient receptor potential melastatin 2 (TRPM2) and nucleotide-binding oligomerization domain, leucine-rich repeat and pyrin domain-containing 3 (NLRP3) expression, while promoting the expansion of regenerative nonclassical monocytes (Ly6C^lo^), exhibiting low TRPM2 and NLRP3 levels. Further validation demonstrated that Sr ions inhibited NLRP3 inflammasome activation in monocytes via blocking TRPM2 expression and calcium influx, leading to reduced pro-inflammatory cytokine (interleukin-1β and interleukin-18) secretion. Meanwhile, a conditioned medium from Sr-SLA-cultured monocytes exerted robust osteogenic potential by markedly facilitating bone marrow mesenchymal stromal cells’ osteogenic differentiation, due to a Sr-reshaped cytokine profile. Moreover, in vivo study corroborated that monocyte depletion impaired osseointegration, underscoring its indispensable role in implant-mediated bone regeneration. Collectively, Sr-SLA implants reprogrammed monocyte subsets via the TRPM2–Ca^2+^–NLRP3 axis, reshaping the early inflammatory microenvironment to enhance osseointegration. This study establishes a cascade linking material properties, early immune response, and bone regeneration, providing an engineerable target for designing immunomodulatory biomaterials.

## Introduction

Metallic implants are widely used to facilitate osseointegration. The early inflammatory response triggered by titanium implants is a double-edged sword. Moderate immune activation facilitates pathogen clearance and initiates bone repair, while excessive or dysregulated inflammation may lead to fibrous encapsulation and even osseointegration failure [1,2]. Monocytes, as a major component of the innate immune system, play an essential role in early inflammatory responses [3]. Implanted biomaterials with alleviated transplant immune rejection are related to less pro-inflammatory monocyte infiltration [4].

Murine monocytes are classified into classical/intermediate (Ly6C^hi^) and nonclassical (Ly6C^lo^) subclusters, analogs respectively of classical/intermediate (CD14^hi^) and nonclassical (CD14^lo^) monocytes in humans [5,6]. Ly6C^hi^ monocytes, also termed inflammatory monocytes, exhibit high phagocytic and pro-inflammatory capacities. In contrast, Ly6C^lo^ monocytes, known as patrolling monocytes, participate in immune surveillance and tissue repair [3,6]. Abnormal expansion of classical monocytes has been confirmed to be closely related to pathological processes such as glucocorticoid-induced bone loss and senile fracture healing disorder [7,8]. It is known that monocyte activation and differentiation may be strongly impacted by the physicochemical characteristics of implanted material surfaces, such as surface energy, chemical composition, and microstructure [9–11]. Therefore, a more thorough comprehension of the mechanisms underlying monocyte activation is required.

Strontium ions (Sr^2+^) have been widely utilized in orthopedic biomaterials due to their unique dual effects of promoting osteogenesis and inhibiting osteoclastic resorption [12–14]. Recent studies have gradually unveiled the osteoimmunomodulatory potential of Sr^2+^ [15–17]. Our previous studies demonstrated that Sr^2+^-modified titanium implants enhanced osteogenesis by forming a favorable immune microenvironment [18–21]. Notably, our recent findings showed that monocytes dominate the local immune cell population in the early postimplantation phase, and their subpopulation distribution is correlated with bone formation efficiency, suggesting their role in reprogramming the osteoimmune microenvironment [21]. Nevertheless, it is still unknown how the local release of Sr^2+^ from biomaterials affects the osseointegration microenvironment by modifying monocyte subcluster differentiation.

Transient receptor potential melastatin 2 (TRPM2) is a nonselective cation channel linking oxidative stress and calcium ion permeation to nucleotide-binding oligomerization domain, leucine-rich repeat and pyrin domain-containing 3 (NLRP3) inflammasome activation [22–24]. The NLRP3 inflammasome is crucial in the innate immune system, which secretes pro-inflammatory cytokines such as interleukin-1β (IL-1β) and interleukin-18 (IL-18) through the activation of caspase-1 and promotes localized inflammatory responses [25]. Studies have reported that TRPM2 mediates Ca^2+^ influx, inducing monocytes to produce the chemokine interleukin 8 (CXCL8), thereby exacerbating inflammatory neutrophil infiltration [26]. An in vitro study observed that Sr-doped biphasic calcium phosphate effectively reduced inflammatory mediator production by monocytes, yet the underlying mechanisms remain unknown [27]. We hypothesized a possible involvement of the TRPM2–Ca^2+^–NLRP3 axis.

**Fig. 1.**
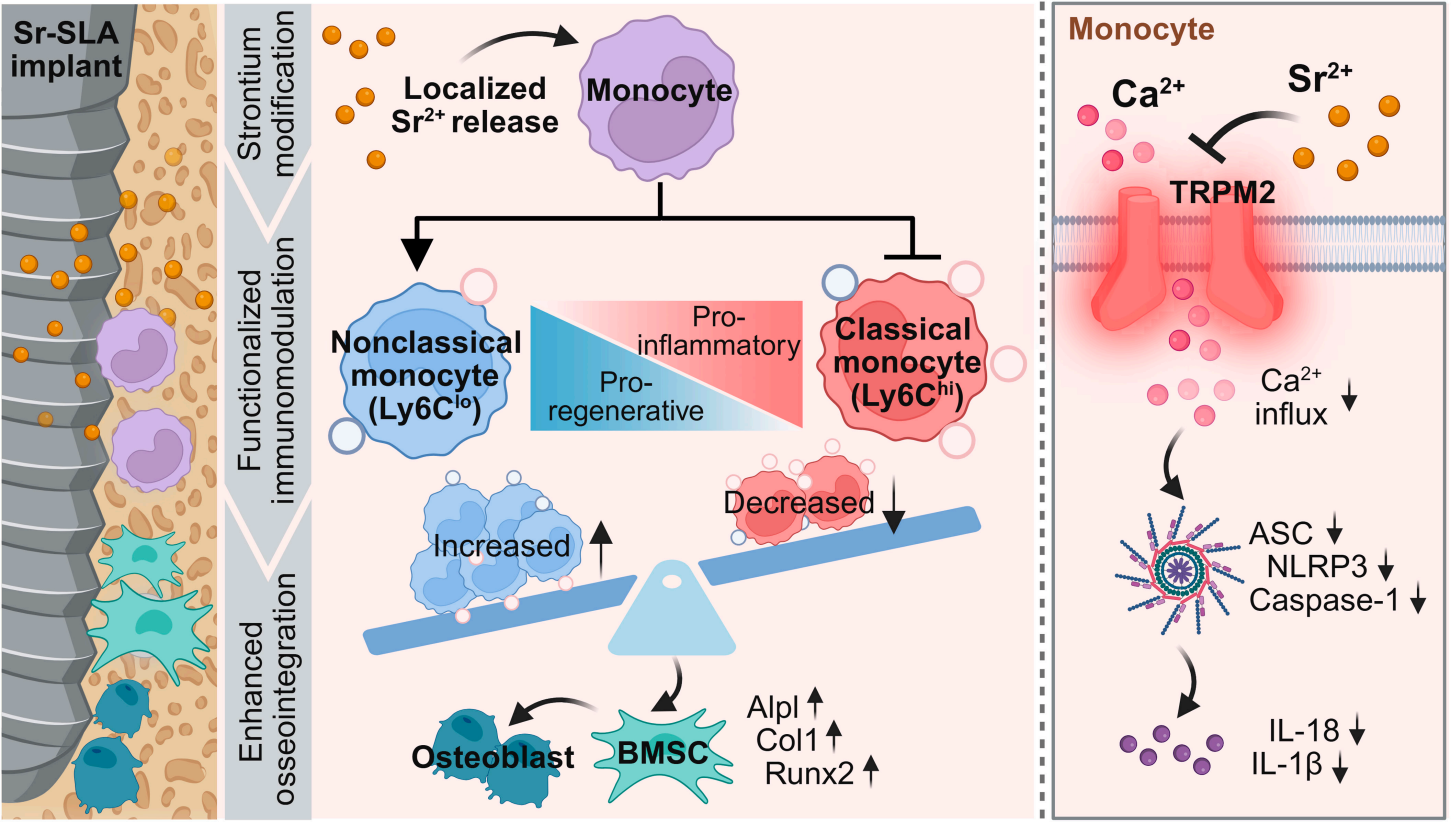
Scheme of monocyte subset reprogramming by a Sr-modified implant via the TRPM2–Ca^2+^–NLRP3 axis. TRPM2, transient receptor potential melastatin 2; NLRP3, nucleotide-binding oligomerization domain, leucine-rich repeat and pyrin domain-containing 3; Sr-SLA, sandblasted, large-grit, acid-etched (SLA) titanium surface incorporated with strontium ions; BMSC, bone marrow mesenchymal stromal cell; ASC, apoptosis-associated speck-like protein containing a CARD; IL-18, interleukin-18; IL-1β, interleukin-1β.

In this study, we engineered sandblasted, large-grit, acid-etched (SLA) titanium surfaces incorporated with strontium ions (Sr-SLA) using hydrothermal treatment. We demonstrated that Sr-SLA implants suppressed pro-inflammatory classical monocyte activation while expanding regenerative nonclassical monocytes, thereby ameliorating early inflammatory microenvironments to enhance osseointegration. Furthermore, the underlying molecular mechanism was associated with the TRPM2–Ca^2+^–NLRP3 axis (Fig. 1). This study not only resolves how functionalized biomaterials precisely regulate monocyte subset dynamics but also provides a theoretical foundation for designing immunomodulatory biomaterials.

## Materials and Methods

### Sample preparation

The surfaces of SLA and Sr-SLA implants were prepared as previously described [28]. Pure titanium samples were provided by Zhejiang Guangci Medical Appliance Co., Ltd, Ningbo, China. The samples for in vivo study were rod shaped, 1 mm in diameter, and 2.5 mm in length, and those for in vitro cell experiments were round slices 28 mm in diameter and 1 mm in thickness or square slices with a side length of 10 mm and a thickness of 1 mm. The samples were divided into 2 groups: (a) SLA: the pure titanium samples were roughened by sandblasting and acid etching with HNO_3_/HF and HCl/H_2_SO_4_. (b) Sr-SLA: the SLA samples were further prepared by hydrothermal treatment with Sr(OH)_2_·8H_2_O solution (99.5% purity, Sigma-Aldrich, USA). All samples were cleaned ultrasonically in deionized water twice for 15 min each time and then dried by nitrogen airflow. The surfaces of the samples were irradiated by ultraviolet light before further experiments.

### Surface characteristics

The surface morphology was observed by scanning electron microscopy (SU70, Hitachi, Japan). The elemental compositions and mappings (Ti and Sr) of the surfaces were determined by energy-dispersive x-ray spectrometry (SU80, Hitachi, Japan). The surface atomic constitution was detected by x-ray photoelectron spectroscopy (Escalab 250Xi, Thermo Fisher Scientific, USA). The crystalline phase of samples was characterized by x-ray diffractometry (XRD-7000, Shimadzu, Japan). For ion release detection of the material surface, 2 materials (round slices with a 30-mm diameter) were immersed in 2 ml of phosphate-buffered saline (PBS) and incubated at 37 °C. The leaching solution at 1, 3, 7, 14, and 21 d was collected, and the concentrations of titanium and strontium ions were detected by inductively coupled plasma mass spectrometry (ICP-MS; XSeries, Thermo Scientific, USA). At least 3 samples were used for the detection of surface characteristics.

### Implantation model

All experiments were approved by the Institutional Animal Care and Use Committee of Zhejiang University, Hangzhou, China (ZJU20250052). C57BL/6 male mice aged 6 to 8 weeks (Zhejiang Academy of Medical Sciences) were used. All animals were randomly divided into 2 groups. Mice were intraperitoneally injected with 10% chloral hydrate (0.33 ml/100 g, Aladdin, C104202) before implantation. After anesthesia, the operative area was swabbed with 5% iodophor and 75% alcohol. A vertical incision was made at the tibia–femoris junction, the soft tissue was removed, and the bone surface of the tibial metaphysis was exposed; 1- and 5-ml syringe needles were prepared to make holes step by step, and SLA or Sr-SLA implants were implanted into the tibia. The skin was sutured after surgery, and penicillin was injected immediately. Samples were collected at 3, 7, and 14 d after surgery for follow-up experiments.

### Monocyte depletion

Clodronate liposomes (CLLs) (15 ml/kg, Clodronate Liposomes, C-010) were intraperitoneally injected the day before and 2 d after surgery. The control group was injected with PBS liposomes (Liposoma, CP-010-010) at the same time point.

### Micro-computed tomography

Mouse tibia containing implants were collected and fixed with 4% paraformaldehyde (PFA; Beyotime, P0099) for 48 h. Analysis was performed using a micro-computed tomography (micro-CT) scanner (MILabs, The Netherlands). The resolution was set to 10 μm. Data reconstruction was completed using the MILabs Rec 10.16 software. Data analysis was performed using the Imalytics Preclinical 2.1 software to obtain bone volume fraction (BV/TV) and trabecular thickness.

### Histological analysis

The fixed mouse tibia was decalcified with 10% ethylenediaminetetraacetic acid (EDTAl Servicebio) for 2 weeks. The implants were carefully removed, and the decalcified tibia was dehydrated with graded ethanol, embedded in paraffin, and cut into 4-μm slices parallel to the long axis of the implants. Sections were stained with hematoxylin and eosin (H&E) to assess the level of new bone formation. For immunohistochemical (IHC) staining, sections were incubated with 3% hydrogen peroxide for 10 min, followed by sodium citrate buffer in an autoclave at 121 °C for 15 min. Subsequently, they were incubated with TRPM2 antibody (HUABIO, HA500437) and NLRP3 antibody (Proteintech, 68102). For immunofluorescence staining, slices were incubated with CD11b antibody (Proteintech, YM8440), lymphocyte antigen 6 family member C (Ly6C) antibody (HUABIO, HA500088), TRPM2 antibody (HUABIO, HA500437), and NLRP3 antibody (Proteintech, 68102) and then were incubated with different fluorescent secondary antibodies. The sections were viewed under a microscope and photographed.

### Specimen harvest for RNA-seq and single-cell RNA-seq

We performed bulk RNA sequencing (RNA-seq) and single-cell RNA sequencing (scRNA-seq) of the mouse implantation model 3 d postsurgery. Bone marrow cells around implants were collected to prepare single-cell suspensions as previously described [20]. The tibia containing the implant was harvested, and the bone marrow cells were flushed out with Hank’s balanced salt solution containing 10% 0.1 μM EDTA. Then, the implants were digested in a mixed-enzyme solution containing 1 mg/ml dispase (Gibco, 17105041), 1 mg/ml collagenase II (Yeasen, 40508ES60), and 0.15 μM DNase I (Thermo Fisher, EN0521) for 30 min at 37 °C. Bone marrow cells and peri-implant cells were filtered with 70-μm cell sieves and centrifuged at 1,500 rpm for 5 min. Red blood cells (RBCs) were then lysed with RBC lysis buffer (Beyotime, C3702). Then, the cells were washed with Hank’s balanced salt solution and filtered with 40-μm cell sieves. Bulk RNA and single-cell RNA library preparations and sequencing were conducted by Gene Denovo Biotechnology Co. (Guangzhou, China).

### Monocyte isolation

C57BL/6 male mice aged 6 to 8 weeks were used for primary mononuclear cell isolation. Bone marrow cells of both the tibia and femur were obtained and centrifuged, and then, RBCs were lysed with RBC lysis buffer (Beyotime, C3702). The cells were cultured in alpha minimum essential medium (αMEM) with 10% fetal bovine serum and 1% penicillin and streptomycin. After 1 d, the suspension cells were collected and cultured with complete αMEM with 10 ng/ml macrophage colony-stimulating factor. The adherent cells were obtained after 3 d.

### Cell Counting Kit-8 assay

Monocytes were seeded into 96-well plates at a density of 1 × 10^4^ cells/well, and 24 h postseeding, the medium was changed to different concentrations of strontium chloride (SrCl_2_), 1,2-dioleoyl-3-trimethylammonium propane (DOTAP chloride; GlpBio, GC41811), and 6,7-dimethoxy-2-(1-piperazinyl)-4-quinazolinamine hydrochloride (DPQ; GlpBio, GC15294). After 12 h cultured, the original medium was replaced with a medium containing 10% Cell Counting Kit (CCK-8) solution (GlpBio, GK10001). After incubation at 37 °C for 2 h, the optical density value at 450 nm was detected by an enzymoleter, and cell viability was counted in each group.

### Quantitative real-time PCR

Total RNA was extracted by TRIzol reagent, and the RNA purity and concentration of the samples were detected by a spectrophotometer (Thermo Fisher Scientific, NanoDrop One). Complementary DNA was synthesized by reverse transcription using PrimeScript RT Master Mix (Takara). Reverse transcription quantitative polymerase chain reaction (PCR) assay (Bio-Rad, CFX384 Real-Time System) was performed using TB Green Premix Ex Taq (Takara) with complementary DNA as template. Primer design, synthesis, and purification were completed by Sangon Biotech (Shanghai, China). The sequences of relevant primers are shown in Table S1. The relative gene expression levels of target genes were normalized to the housekeeping gene GAPDH.

### Western blot

The cells were lysed with radio immunoprecipitation assay lysis buffer (Beyotime, P0013B) and then centrifuged at 12,000 rpm at 4 °C for 15 min, and the supernatant was collected. A bicinchoninic acid protein assay kit (Beyotime, P0010) was used to determine the protein concentration of the sample. Protein loading buffer and PBS were used to quantify protein samples. An equal amount of protein samples was loaded onto sodium dodecyl sulfate–polyacrylamide gel electrophoresis gel and transferred to a polyvinylidene fluoride membrane (Millipore, IPVH00010). After blocking with a high-efficiency blocking solution (Genefist, GF1915), the membrane was incubated with TRPM2 antibody (HUABIO, HA500437), NLRP3 antibody (Cell Signaling Technology, 15101), caspase-1 antibody (Cell Signaling Technology, 24232), and cleaved caspase-1 antibody (Cell Signaling Technology, 89332) at 4 °C overnight. Then, the membrane was incubated with goat anti-rabbit secondary antibody (Cell Signaling Technology, 7074) at room temperature for 1 h. The immunoreactive bands were developed with enhanced chemiluminescence solution (MCE, HY-K1005) and visualized by a chemiluminescent imager.

### Immunofluorescent staining

Monocytes were seeded into a 24-well plate at a density of 1 × 10^5^ cells per well. After 24 h, monocytes were fixed with 4% PFA for 20 min and washed with PBS. Then, immunostaining permeable solution (Beyotime, P0096) was used to break the cell membrane for 5 min, and the cells were blocked by immunostaining QuickBlock (Beyotime, P0260) for 10 min. The cells were incubated with TRPM2 antibody (HUABIO, HA500437) and NLRP3 antibody (Proteintech, 68102) at 4 °C overnight and then incubated with Goat Anti-Rabbit IgG H&L (Alexa Fluor 488) (Abcam, ab150077) and Goat Anti-Mouse IgG H&L (Alexa Fluor 647) (Abcam, ab150115) at room temperature for 1 h. The nuclei were stained with 4′,6-diamidino-2-phenylindole (Beyotime, C1006) for 5 min. The cells were observed with a laser confocal scanning microscope (Nikon A1, Japan). The Image Pro Plus 6.0 software was used for semiquantitative analysis.

### Flow cytometry

Single-cell suspensions were prepared from tibial bone marrow, and cells were cultured on material surfaces. The cell suspension was filtered through a 40-μm cell strainer to remove debris. RBCs were lysed using RBC lysis buffer (Beyotime, C3702), followed by washing with cell staining buffer. Cells were incubated with Mouse BD Fc Block (BD Biosciences, 553141) at 4 °C for 30 min to prevent nonspecific antibody binding. After centrifugation, cells were resuspended in antibody cocktails containing PE Anti-mouse/human CD11b Antibody (BioLegend, 101207), APC Anti-mouse Ly-6G Antibody (BioLegend, 127614), and Alexa Fluor 488 Anti-mouse Ly-6C Antibody (BioLegend, 128022). Cells were incubated with antibodies at 4 °C in the dark for 30 min. Then, cells were washed twice with cell staining buffer to remove unbound antibodies. Finally, stained cells were analyzed using a CytoFLEX LX flow cytometer (Beckman Coulter, USA). Data were processed and visualized using the FlowJo software (version 10.8.1, BD Biosciences).

### Calcium ion detection

For the detection of calcium accumulation, intracellular calcium ions were labeled using the Fluo-4 AM probe (Beyotime, S1060). The cells were incubated at 37 °C without light for 30 min, washed with PBS, and then inoculated with materials, and the optical density value at a 488-nm wavelength was detected by an enzymoleter at different time points. For the detection of calcium transient, cells were seeded into confocal dishes, incubated at 37 °C for 30 min in the dark with the Fluo-4 AM probe, then washed with PBS, and treated under different conditions. At the same time, a Leica confocal microscope (Leica TCS SP8, Germany) was used to dynamically monitor the changes in intracellular calcium ions, and images were acquired at 5-s intervals over a 5-min period. The fluorescence intensity of intracellular calcium ions was analyzed by the LAS X software.

### Enzyme-linked immunosorbent assay

After 24 h of culturing monocytes on implant surfaces, the extracts were collected and stored at −80 °C. The levels of IL-1β and IL-18 were detected according to the instructions in the enzyme-linked immunosorbent assay (ELISA) detection kit: IL-1β (ELK Biotechnology, ELK1271) and IL-18 (ELK Biotechnology, ELK2269). The absorbance was detected with a microplate reader. The protein levels of IL-1β and IL-18 were calculated using the absorbance value of standard samples.

### BMSC isolation

C57BL/6 male mice aged 6 to 8 weeks were used for primary bone marrow mesenchymal stromal cell (BMSC) isolation. Bone marrow cells of both the tibia and femur were obtained and centrifuged, and then RBCs were lysed with RBC lysis buffer (Beyotime, C3702). The cells were cultured in complete αMEM. After 24 h, the medium was replaced to remove nonadherent cells. The medium was then changed every 3 d. The cells at passage 2 were used for follow-up experiments.

### Osteogenic differentiation induction

The leach solution of the material and the supernatant of monocytes cultured from the material were collected and used as a conditioned medium for further culture of BMSCs. For osteogenic differentiation induction, BMSCs were seeded into a 24-well plate at a density of 5 × 10^4^ cells per well. The complete αMEM was supplemented with ascorbic acid (50 ng/ml), β-glycerophosphate (10 mM), and dexamethasone (100 nM). The medium was then changed every 3 d.

### Alkaline phosphatase activity

After 7 and 14 d of osteogenic induction, alkaline phosphatase (ALP) staining was performed according to the instructions of the BCIP/NBT Alkaline Phosphatase Color Development Kit (Beyotime, C3206). BMSCs were fixed with 4% PFA for 20 min and washed with PBS. Then, ALP staining solution was added and the cells were stained for 30 min at room temperature. The ALP staining of cells in each well was recorded by a camera. Cell lysis buffer was added to each well, the cells were lysed on ice for 10 min, and the supernatant was collected and centrifuged. According to the instructions of the ALP kit (Nanjing Jiancheng Bioengineering Institute, A059-2), the absorbance at 520 nm was determined. Then, a bicinchoninic acid protein assay kit (Beyotime, P0010) was used to detect the protein concentration of the sample, and the normalized ALP relative activity was calculated.

### Alizarin Red staining

After 7 and 14 d of osteogenic induction, BMSCs were fixed with 4% PFA for 20 min and washed with PBS. Alizarin Red staining (ARS) solution (OriCell, ALIR-10001) was added, and the cells were stained for 30 min at room temperature. After washing with PBS, the ARS of cells in each well was recorded by a camera. For quantitative analysis, 500 μl of 10% cetylpyridinium chloride solution (Aladdin) was added to each well, and the wells were shaken and incubated at 37 °C for 30 min. The absorbance value of the supernatant at 562 nm was detected by a microplate reader.

### Statistical analysis

Statistical analysis was performed using GraphPad Prism software version 10.0. Data are expressed as mean ± standard deviation. Data between 2 groups were compared using the *t* test, and data among the multiple groups were compared using one-way analysis of variance with the Tukey post hoc test. *P* < 0.05 was considered statistically significant (*P < 0.05, **P < 0.01, ***P < 0.001, and ****P < 0.0001).

## Results

### Sr-SLA implants ameliorate early inflammatory microenvironments and promote osteogenesis

**Fig. 2.**
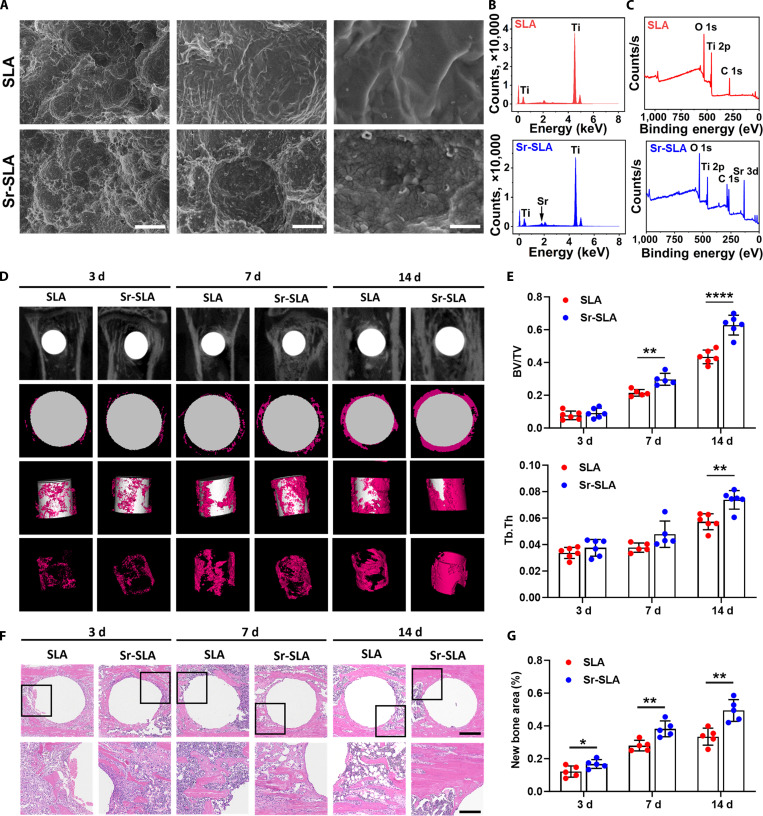
Sr-SLA implants promoted bone formation. (A) Representative scanning electron microscopy (SEM) images of SLA and Sr-SLA surfaces (scale bars = 5 μm, 1 μm, and 250 nm). (B) Energy-dispersive x-ray spectrometry (EDS) spectra of SLA and Sr-SLA surfaces. (C) X-ray photoelectron spectroscopy (XPS) patterns of SLA and Sr-SLA surfaces. (D) Micro-computed tomography (micro-CT) evaluation of bone regeneration in the SLA and Sr-SLA groups at 3, 7, and 14 d after implantation (the pink area indicates new bone formation). *n* = 6 mice per group and per time point. (E) Bone volume fraction (BV/TV) and trabecular thickness (Tb.Th) of regenerated tissues surrounding implants at 3, 7, and 14 d after implantation in each group. (F) Hematoxylin and eosin (H&E) staining of paraffin sections in the SLA and Sr-SLA groups at 3, 7, and 14 d after implantation (scale bar = 500 μm at low magnification, scale bar = 200 μm at high magnification). *n* = 5 mice per group and per time point. (G) New bone area of regenerated tissues surrounding implants at 3, 7, and 14 d after implantation in each group. *P < 0.05; **P < 0.01; ****P < 0.0001.

To probe the potential roles of Sr^2+^ in early bone healing, we generated Sr^2+^-doped titanium implants and subsequently implanted them into murine tibiae. The physicochemical properties of SLA and Sr-SLA were characterized. Scanning electron microscopy revealed similar honeycomb-like macrostructures in both groups, and Sr-SLA surfaces displayed uniformly distributed nanoscale particles (Fig. 2A). Energy-dispersive x-ray spectrometry and elemental mapping confirmed homogeneous strontium distribution on Sr-SLA surfaces (Fig. 2B and Fig. S1A). X-ray photoelectron spectroscopy detected titanium peaks in both groups, with additional strontium peaks observed on Sr-SLA surfaces (Fig. 2C). X-ray diffractometry further validated strontium incorporation into Sr-SLA surfaces (Fig. S1B). In vitro ion release assays indicated an initial high release of Sr^2+^ during the first day, followed by a slower sustained release up to 21 d (Fig. S2A). During the period analyzed, there was no statistical difference in titanium ion release between the 2 groups (Fig. S2B).

To evaluate osteogenic properties, SLA and Sr-SLA implants were implanted into murine tibiae. Micro-CT analysis revealed no significant difference in new bone formation 3 d postimplantation, but Sr-SLA implants exhibited higher bone volumes at 7 and 14 d (Fig. 2D and E). H&E staining results also confirmed the pro-osteogenic property of Sr-SLA (Fig. 2F and G). The detection of serum Sr^2+^ concentration showed no intergroup difference (Fig. S3), indicating that the localized rather than systemic effects of Sr^2+^ released from Sr-SLA implants.

**Fig. 3.**
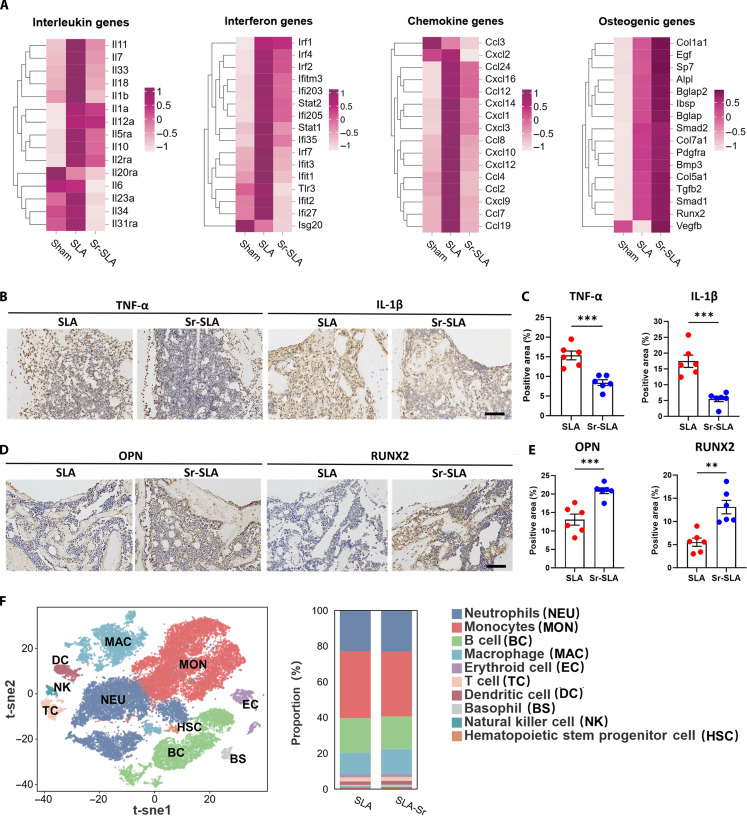
Sr-SLA implants improved the early stage of inflammatory microenvironment postimplantation. (A) Transcriptomic analysis of the tissues surrounding the SLA and Sr-SLA implants 3 d after implantation showed the expression levels of pro-inflammatory cytokines and osteogenic genes in each group. The sham group served as a blank control without any implanted materials. (B) Immunohistochemical (IHC) staining of tumor necrosis factor-α (TNF-α) and IL-1β (brown) 3 d postimplantation (scale bar = 100 μm). (C) Semiquantitative analysis of the immunohistochemistry staining of TNF-α and IL-1β. (D) Immunohistochemistry staining of osteopontin (OPN) and runt-related transcription factor 2 (RUNX2) (brown) 3 d postimplantation (scale bar = 100 μm). (E) Semiquantitative analysis of the immunohistochemistry staining of OPN and RUNX2. (F) Single-cell RNA sequencing (scRNA-seq) of the regenerated tissue surrounding SLA and Sr-SLA implants 3 d after surgery. The t-distributed stochastic neighbor embedding (tSNE) plot shows the distribution of total cells divided into 10 cell types. Barplots showing the proportions of the 10 cell types between the 2 groups. **P < 0.01; ***P < 0.001.

Transcriptomic profiling of peri-implant bone tissues 3 d postimplantation revealed a less inflammatory and pro-osteogenic microenvironment in the Sr-SLA group, characterized by the down-regulation of pro-inflammatory cytokines and the up-regulation of osteogenic genes (Fig. 3A). IHC staining confirmed decreased expressions of IL-1β and TNF-α along with increased expressions of osteopontin (OPN) and runt-related transcription factor 2 (RUNX2) in the Sr-SLA group (Fig. 3B to E). To further comprehensively explore the differences in the osteoimmune microenvironment surrounding SLA and Sr-SLA implants, we performed scRNA-seq on peri-implant tissues harvested 3 d postimplantation. Ten main cell types were identified according to classic marker genes (Fig. 3F), namely, monocytes, neutrophils, macrophages, B cells, T cells, dendritic cells, erythroid cells, basophils, natural killer cells, and hematopoietic stem progenitor cells. Among them, monocytes constituted the largest proportion in both groups (Table S2). The functional annotation of monocytes revealed a strong association with immune and inflammatory responses (Fig. S4), suggesting their pivotal role in the early inflammatory phase of implant-mediated osseointegration.

### Sr-SLA implants mediate the reprogramming of monocyte subset homeostasis to reshape a pro-regenerative immune microenvironment

**Fig. 4.**
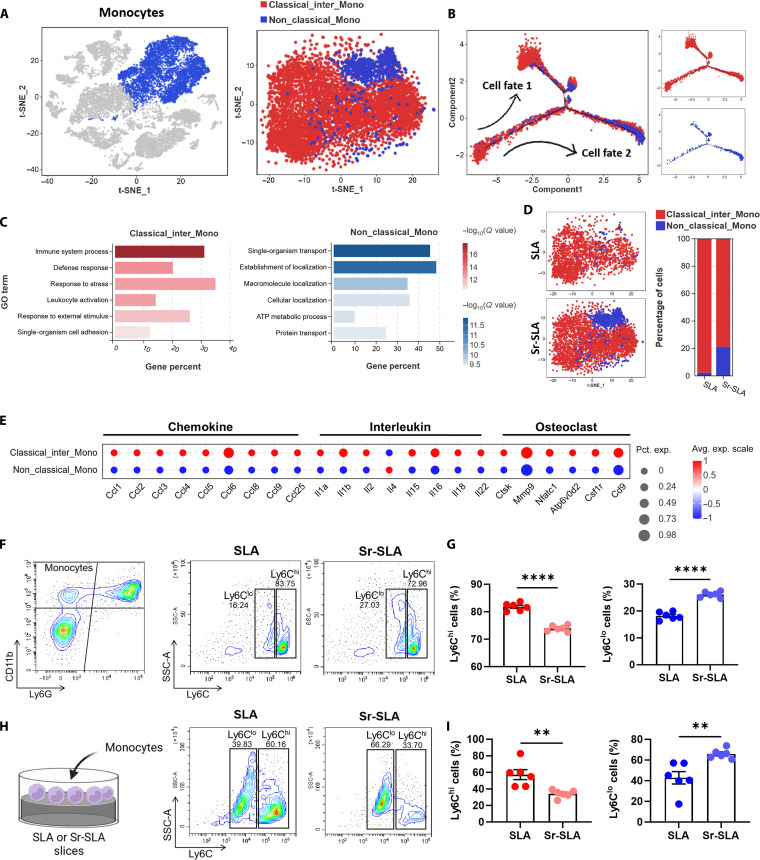
Sr-SLA implants drove monocyte differentiation into the nonclassical subset. (A) tSNE plot of the 2 subclusters in monocytes. (B) Trajectory of the differentiation of monocytes predicted by Monocle 2. (C). Barplot showing the Gene Ontology (GO) enrichment of monocyte subclusters. (D) tSNE plot and barplot showing the percentage of the 2 subclusters of monocytes in the SLA and Sr-SLA groups. (E) Dot plot showing the expression levels of chemokine, interleukin, and osteoclast genes in monocyte subclusters. (F and G) Flow cytometry analysis of Ly6C^hi^ and Ly6C^lo^ cells gated on CD11b^+^Ly6G^−^ cells in the SLA and Sr-SLA groups 3 d after implantation. *n* = 6 mice per group. (H and I) Flow cytometry analysis of the Ly6C^hi^ and Ly6C^lo^ cells of monocytes cultured on SLA and Sr-SLA material surfaces for 24 h. **P < 0.01; ****P < 0.0001. Pct. exp., percent expressed; Avg. exp., average expression; SSC-A, side scatter area; Ly6C, lymphocyte antigen 6 family member C.

To elucidate the effect of strontium incorporation on monocyte and biological functions, monocytes were subclustered into Ly6C^hi^ classical and Ly6C^lo^ nonclassical monocytes (Fig. 4A and Fig. S5A). Pseudotime trajectory analysis revealed bifurcated differentiation pathways (Fig. 4B). Gene Ontology enrichment demonstrated that classical monocytes were related to immune system processes and defense responses, whereas nonclassical monocytes were associated with organic substance transport and molecular localization (Fig. 4C). The gene expression heatmap showed classical monocytes up-regulated pro-inflammatory-cytokine- and osteoclast-related genes, while nonclassical monocytes displayed anti-inflammatory properties with reduced interferon-induced genes (Fig. 4E and Fig. S5B). The proportion of nonclassical monocytes was substantially higher around Sr-SLA implants than that in the SLA group (21.21% vs. 2.28%), while classical monocytes were reduced (78.79% vs. 97.72%) (Fig. 4D). In vivo flow cytometry confirmed a lower proportion of Ly6C^hi^ classical monocytes and a higher portion of Ly6C^lo^ nonclassical monocytes in the Sr-SLA group (Fig. 4F and G). It was further supported by in vitro flow cytometry results (Fig. 4H and I). These findings indicated that Sr-SLA implants critically modulated Ly6C^hi^/Ly6C^lo^ monocyte homeostasis, potentially reshaping the inflammatory microenvironment.

### Sr-SLA implants suppress NLRP3 inflammasome activation in monocytes

**Fig. 5.**
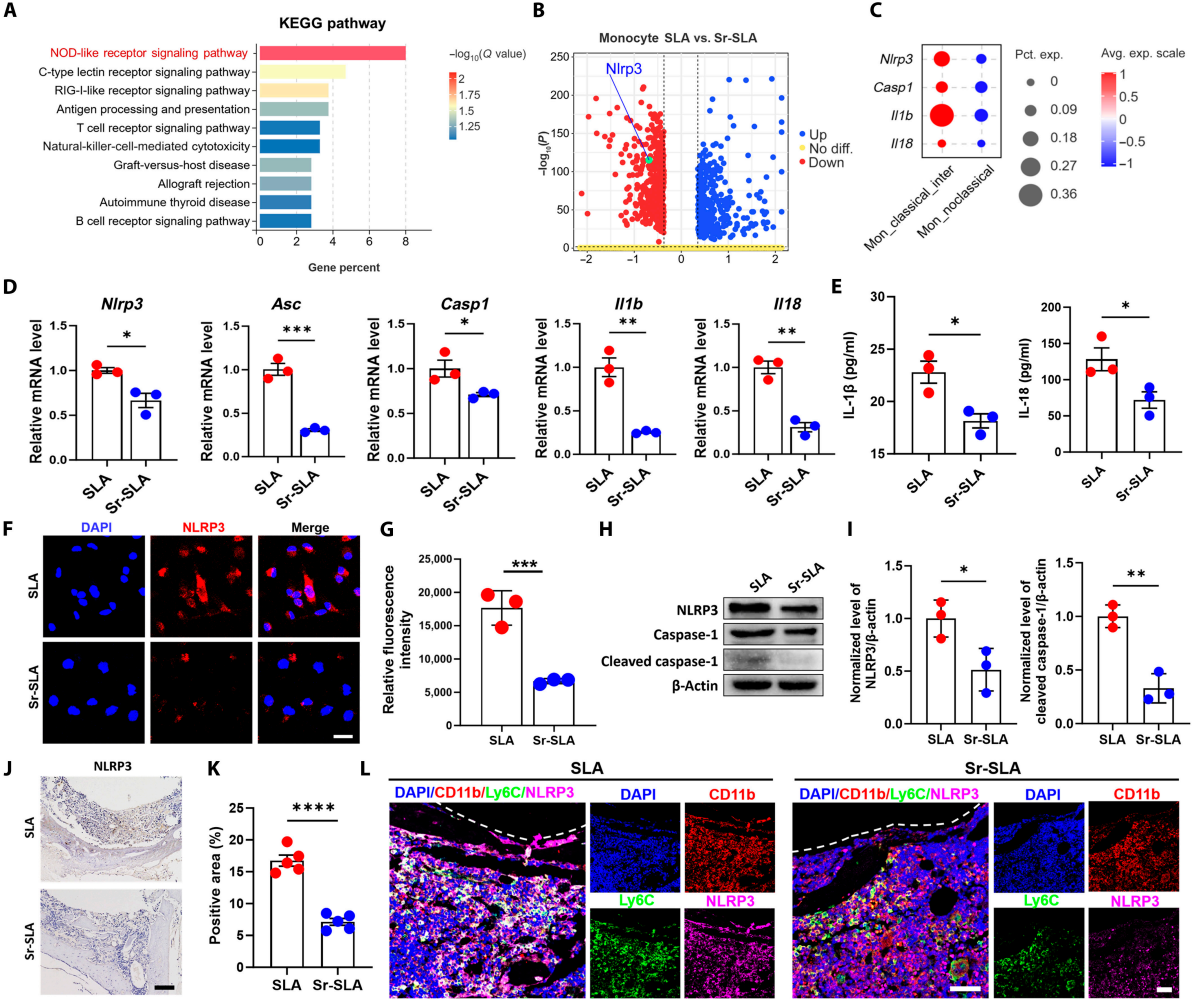
Sr-SLA implants suppressed NLRP3 inflammasome activation in monocytes. (A) Barplot showing the Kyoto Encyclopedia of Genes and Genomes (KEGG) pathway of the down-regulated genes of monocytes in the Sr-SLA group. (B) Volcano plot showing the expression level of Nlrp3 in monocytes between the SLA and Sr-SLA groups. (C) Dot plot showing the expression levels of inflammasome genes for monocyte subclusters. (D) Quantitative real-time polymerase chain reaction (RT-qPCR) results of the inflammasome gene expression levels of monocytes cultured on SLA and Sr-SLA material surfaces for 12 h. (E) Enzyme-linked immunosorbent assay (ELISA) showing the levels of IL-1β and IL-18 in the monocyte culture supernatant. (F and G) Immunofluorescent (IF) staining and semiquantitative analysis results of the NLRP3 expression level of monocytes cultured on SLA and Sr-SLA material surfaces for 24 h (scale bar = 20 μm). (H and I) Western blot (WB) and quantitative analysis of the NLRP3, caspase-1, cleaved caspase-1, and β-actin of monocytes cultured on SLA and Sr-SLA material surfaces for 24 h. (J and K) IHC staining and semiquantitative analysis results of NLRP3 (brown) 3 d postimplantation (scale bar = 100 μm). (L) Co-immunostaining of the CD11b, Ly6C, and NLRP3 expression of paraffin sections in the SLA and Sr-SLA groups 3 d after implantation (scale bar = 50 μm). *P < 0.05; **P < 0.01; ***P < 0.001; ****P < 0.0001. mRNA, messenger RNA; DAPI, 4′,6-diamidino-2-phenylindole.

The Kyoto Encyclopedia of Genes and Genomes (KEGG) enrichment analysis of differentially expressed genes between the Sr-SLA group and the SLA group revealed the down-regulation of the nucleotide oligomerization domain (NOD)-like receptor signaling pathway in the monocytes of the Sr-SLA group (Fig. 5A). Specifically, the expression of Nlrp3 in monocytes was reduced in the Sr-SLA group (Fig. 5B), and genes associated with the NLRP3 inflammasome showed lower expression levels in nonclassical monocytes than in classical monocytes (Fig. 5C). NLRP3 inflammasome activation is a central mediator for the production of mature IL-1β [22]. To determine whether Sr-SLA implants suppress monocyte polarization through NLRP3 inflammasomes, we employed a combination of in vitro and in vivo experiments. In vitro, the gene expression of *Nlrp3*, *Asc*, *Casp1*, *Il1b*, and *Il18* was decreased in primary monocytes 12 h after culturing on the Sr-SLA surface (Fig. 5D). The ELISA results also revealed that IL-1β and IL-18 secretion decreased in the medium (Fig. 5E). Immunofluorescent (IF) staining showed diminished NLRP3 fluorescence intensity of monocytes on the Sr-SLA surface (Fig. 5F and G). In addition, Western blot (WB) confirmed reduced NLRP3, caspase-1, and cleaved caspase-1 protein levels in the Sr-SLA group (Fig. 5H and I). In vivo, SLA implants exhibited higher NLRP3-positive cell counts (Fig. 5J and K), with multiplex immunofluorescence staining showing more CD11b^+^Ly6C^+^NLRP3^+^ cells (Fig. 5L).

### The TRPM2/Ca^2+^ signal regulates Sr-mediated NLRP3 inflammasome inhibition in monocytes

**Fig. 6.**
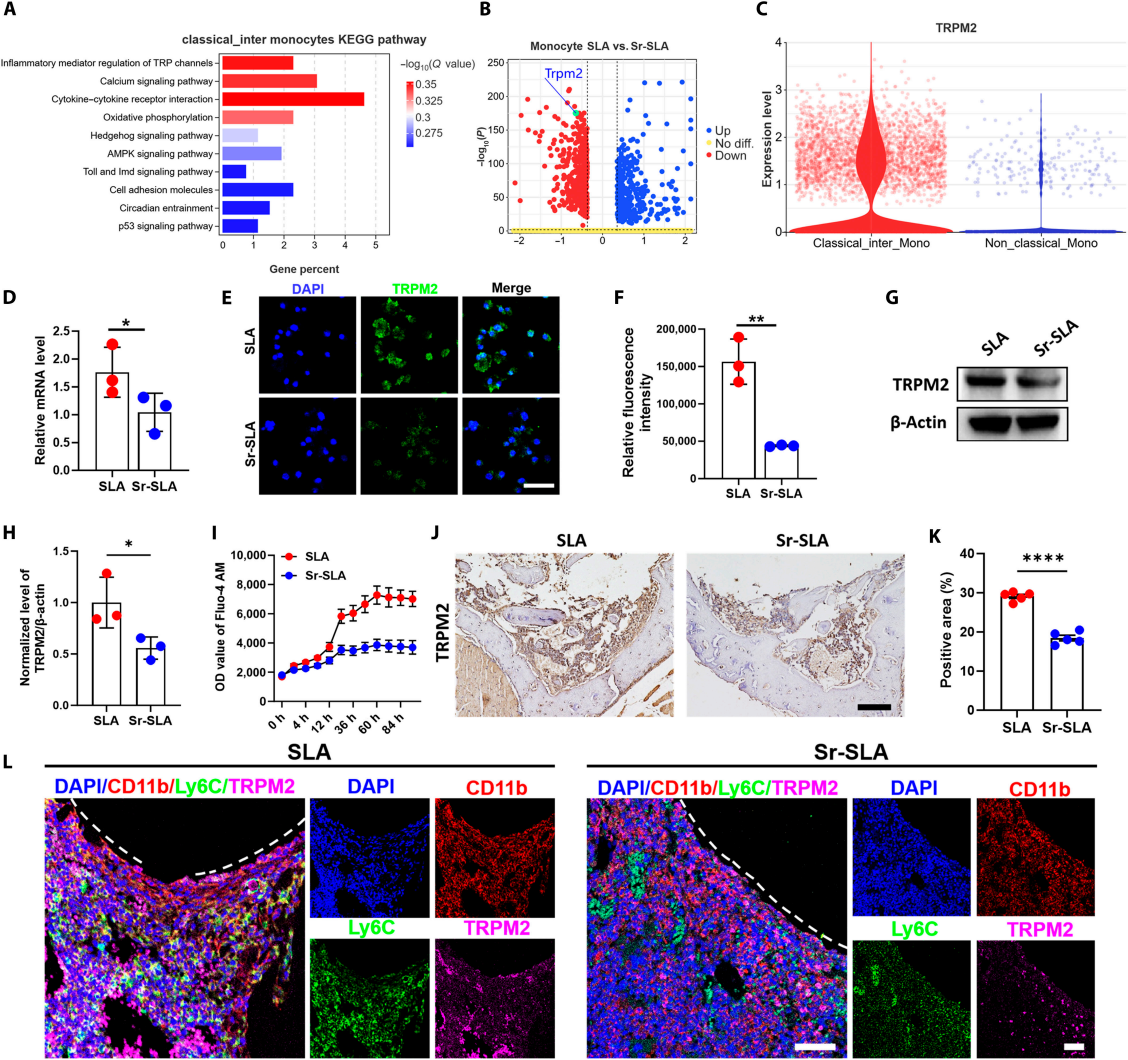
Sr-SLA implants regulated monocytes through TRPM2/Ca^2+^ signals. (A) Barplot showing the KEGG pathway from the up-regulated genes of classical/intermediate monocytes. (B) Volcano plot showing the expression level of Trpm2 in monocytes between the SLA and Sr-SLA groups. (C) Violin plot showing the expression levels of Trpm2 for monocyte subclusters. (D) RT-qPCR results of the *Trpm2* gene expression level of monocytes cultured on SLA and Sr-SLA material surfaces for 12 h. (E and F) IF staining and semiquantitative analysis results of the TRPM2 expression level of monocytes cultured on SLA and Sr-SLA material surfaces for 24 h (scale bar = 50 μm). (G and H) WB and quantitative analysis of the TRPM2 and β-actin of monocytes cultured on SLA and Sr-SLA material surfaces for 24 h. (I) The intracellular Ca^2+^ level of monocytes was detected by Fluo-4 AM fluorescence staining. (J and K) IHC staining and semiquantitative analysis results of TRPM2 (brown) 3 d postimplantation (scale bar = 100 μm). (L) Co-immunostaining of the CD11b, Ly6C, and TRPM2 expression of paraffin sections in the SLA and Sr-SLA groups 3 d after implantation (scale bar = 50 μm). *P < 0.05; **P < 0.01; ****P < 0.0001. OD, optical density.

KEGG analysis revealed up-regulated transient receptor potential (TRP) channels in classical/intermediate monocytes. (Fig. 6A). Among TRP calcium channels, TRPM2 showed the highest expression in monocytes (Fig. S6). The volcano plots showed lower Trpm2 expression in the Sr-SLA group (Fig. 6B). Nonclassical monocytes expressed less Trpm2 than classical/intermediate monocytes (Fig. 6C). Quantitative real-time polymerase chain reaction (RT-qPCR), IF staining, and WB confirmed reduced messenger RNA and protein levels of TRPM2 in Sr-SLA-treated monocytes (Fig. 6D to H). The Fluo-4 AM fluorescent probe was used to label intracellular calcium ions in monocytes. The concentration of intracellular calcium ions in monocytes on the SLA surface was higher than that in the Sr-SLA group over time (Fig. 6I), indicating that the Sr-SLA material inhibited intracellular calcium ions in monocytes. According to IHC results, a higher number of TRPM2-positive cells were observed around SLA implants 3 d after surgery (Fig. 6J and K). Multiple immunofluorescence staining observed more CD11b^+^Ly6C^+^TRPM2^+^ cells around SLA implants than those in the Sr-SLA group (Fig. 6L).

**Fig. 7.**
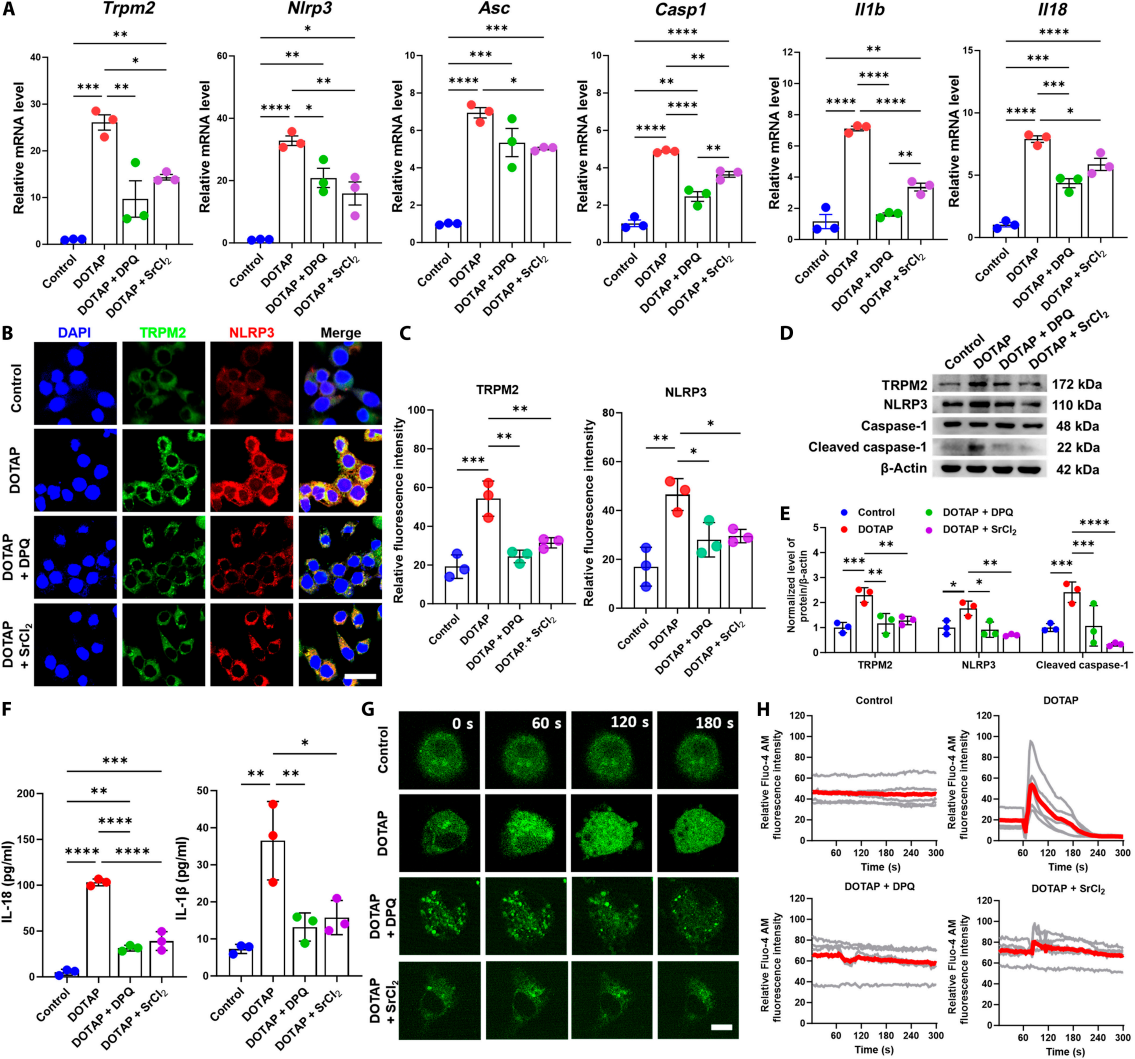
Strontium ions exhibited the same effect as the TRPM2 inhibitor. (A) RT-qPCR results of *Trpm2* and the inflammasome gene expression levels of monocytes cultured under 4 conditions for 12 h. (B and C) IF staining and semiquantitative analysis results of the TRPM2 and NLRP3 expression levels of monocytes cultured under 4 conditions for 24 h (scale bar = 20 μm). (D and E) WB and quantitative analysis of the TRPM2, NLRP3, and β-actin of monocytes cultured under 4 conditions for 24 h. (F) ELISA showing the levels of IL-1β and IL-18 in the monocyte culture supernatant. (G and H) Intracellular calcium dynamics were monitored using the Fluo-4 AM fluorescence probe in live cells. Time-lapse imaging was conducted at 3-s intervals over 5 min (scale bar = 5 μm). *P < 0.05; **P < 0.01; ***P < 0.001; ****P < 0.0001. DOTAP, 1,2-dioleoyl-3-trimethylammonium propane; DPQ, 6,7-dimethoxy-2-(1-piperazinyl)-4-quinazolinamine hydrochloride.

To determine whether Sr^2+^ plays a role in suppressing TRPM2 expression in monocytes, we stimulated monocytes with DOTAP and co-treated them with either the TRPM2 inhibitor DPQ or SrCl_2_. Through CCK-8 assays, the optimal experimental concentrations were determined as 0.5 μg/ml for DOTAP and 200 μM for DPQ. Based on a comprehensive evaluation of CCK-8 results and Sr^2+^ release data from ICP-MS, a concentration of 100 μg/ml was selected for SrCl_2_ (Fig. S7). DOTAP stimulation markedly up-regulated the gene expressions of *Trpm2*, *Nlrp3*, *Asc*, *Casp1*, *Il1b*, and *Il18*, while SrCl_2_ and DPQ exhibited comparable effects in reversing this up-regulation (Fig. 7A). IF and WB results revealed enhanced TRPM2 and NLRP3 protein levels in DOTAP-stimulated monocytes, which were attenuated by co-treatment with SrCl_2_ or DPQ (Fig. 7B to E). Furthermore, a positive correlation was observed between TRPM2 and NLRP3 expression levels (Fig. S8). ELISAs confirmed that SrCl_2_ and DPQ reduced DOTAP-induced secretion of IL-1β and IL-18 in monocyte supernatants (Fig. 7F). To explore the effect of TRPM2 expression on intracellular Ca^2+^ concentrations, live-cell imaging using Fluo-4 AM showed that SrCl_2_ and DPQ stabilized DOTAP-induced Ca^2+^ fluctuations (Fig. 7G and H).

### The Sr-reshaped cytokine profile of monocytes promotes BMSC osteogenesis

**Fig. 8.**
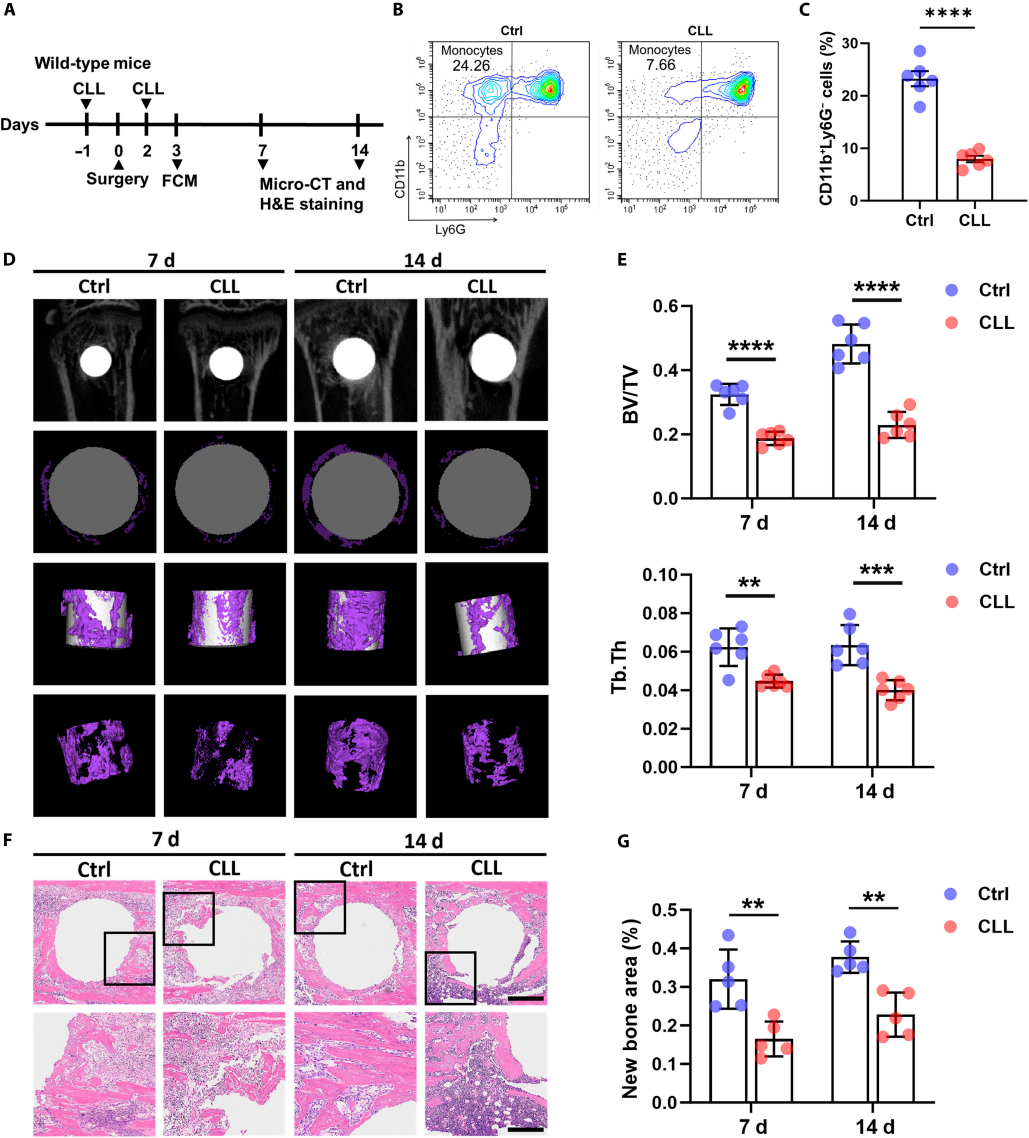
Monocyte depletion impaired new bone formation. (A) Workflow of the experiments on monocyte depletion. (B) Flow cytometry analysis of cells stained by CD11b and Ly6G in the control and clodronate liposome (CLL) groups. *n* = 6 mice per group. (C) Statistical analysis of CD11b^+^Ly6G^−^ cells in the control and CLL groups. (D) Micro-CT evaluation of bone regeneration in control and CLL groups at 7 and 14 d after implantation (the purple area indicates new bone formation). *n* = 6 mice per group and per time point. (E) BV/TV and Tb.Th of regenerated tissues surrounding implants 7 and 14 d after implantation in each group. (F) H&E staining of paraffin sections in the control and CLL groups 7 and 14 d after implantation (scale bar = 500 μm at low magnification, scale bar = 200 μm at high magnification). *n* = 5 mice per group and per time point. (G) New bone area of regenerated tissues surrounding implants 7 and 14 d after implantation in each group. **P < 0.01; ***P < 0.001; ****P < 0.0001. FCM, flow cytometry.

To validate monocytes’ role in implant-mediated bone formation, we intraperitoneally injected CLLs to deplete circulating monocytes. Consistent with previous studies [29], this intervention resulted in the depletion of the majority of CD11b^+^Ly6G^−^ monocytes 3 d after injection. Subsequent analyses of samples collected at 7 and 14 d demonstrated markedly impaired new bone formation around implants in monocyte-depleted mice, as evidenced by micro-CT and H&E staining (Fig. 8). These results indicate that monocytes play a crucial role in implant-mediated bone formation.

**Fig. 9.**
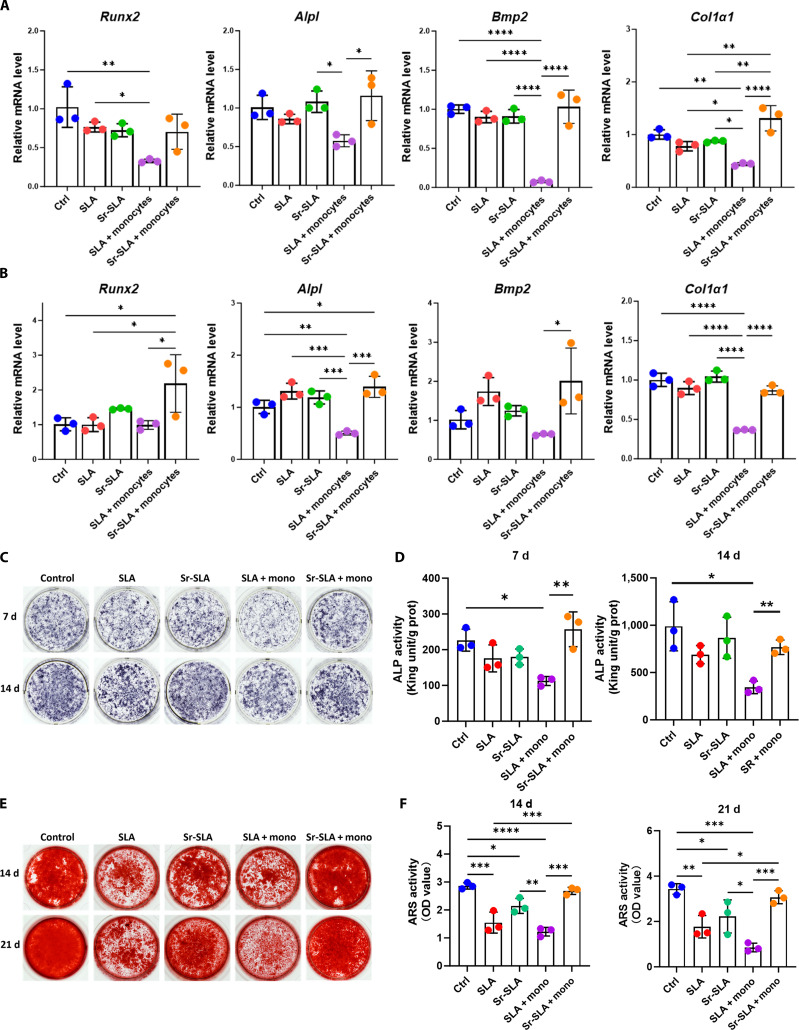
Strontium-modulated monocytes promoted BMSC osteogenesis. (A and B) RT-qPCR results of osteogenic-differentiation-related gene expression levels after 7 and 14 d of induction. (C and D) Alkaline phosphatase (ALP) staining and ALP activity measurement of BMSCs after induction for 7 and 14 d. (E and F) Alizarin Red staining (ARS) and quantitative analysis results of BMSCs after 14 and 21 d of induction. *P < 0.05; **P < 0.01; ***P < 0.001; ****P < 0.0001.

The leach solution of SLA and Sr-SLA materials and the supernatant of monocytes cultured on these materials were collected as a conditioned medium to treat mouse primary BMSCs. RT-qPCR analysis revealed that at 7 and 14 d, the supernatant from Sr-SLA-treated monocytes substantially enhanced the expression of osteogenic genes (*Runx2*, *Alpl*, *Bmp2*, and *Col1a1*) in BMSCs compared to the supernatant from SLA-treated monocytes, with this difference being more obvious than that between pure Sr-SLA and SLA material leach solution (Fig. 9A and B). ALP staining demonstrated that BMSCs treated with the supernatant of monocytes cultured with Sr-SLA exhibited the highest ALP activity, showing statistically significant differences compared to the supernatant group of SLA-treated monocytes (Fig. 9C and D). In ARS experiments at 14 and 21 d, the supernatant from Sr-SLA-cultured monocytes induced substantially more mineralized nodule formation in BMSCs than the SLA group (Fig. 9E and F). These results indicated that the Sr-SLA-mediated monocyte culture supernatant could markedly promote the osteogenic differentiation of BMSCs compared with the extraction liquid of pure material, which was attributed to Sr-SLA regulating the proportion of monocyte subsets, leading to a reduction in pro-inflammatory cytokines.

## Discussion

There is an increasing amount of research exploring the impact of immune cells on bone healing. However, monocytes, which are early responders following biomaterial implantation, remain less explored. In this work, Sr-incorporated Ti-based implants were shown to enhance osseointegration, as reported in previous studies [20,21]. Further, we demonstrated that Sr-SLA implants reprogrammed monocyte subsets via the TRPM2–Ca^2+^–NLRP3 axis, reshaping the early inflammatory microenvironment to enhance osseointegration by reducing pro-inflammatory cytokine secretion. Therefore, this study provides new perspectives for the immunomodulatory role of Sr-functionalized biomaterials on monocytes and the underlying mechanism by which Sr^2+^ influences the monocyte phenotype. The results offer a novel strategy for surface-functionalized biomaterial design.

Monocytes, under pathological conditions, are rapidly recruited to inflammatory sites, bidirectionally modulating tissue repair via pro- and anti-inflammatory functions [3,6]. In a titanium-implant- and hydrogel-mediated tissue regeneration microenvironment, monocytes account for a large proportion [30,31]. Another study reported that monocyte knockout exhibited impaired vascularization and delayed fracture healing in mice [32]. Similarly, our findings revealed that monocytes dominated the early immune infiltrate following titanium implant placement, and their depletion markedly impaired new bone formation, confirming their role as upstream regulators in implant-mediated osteogenesis. The physicochemical characteristics of biomaterials can substantially influence the activation and differentiation of monocytes. For instance, titanium-based materials with reduced surface energy and decreased contact time can diminish the oxidative stress response of monocytes [10]. Zinc silicate/nano-hydroxyapatite/collagen scaffolds can activate the p38 MAPK pathway in monocytes through the release of Zn^2+^/Si^4+^, thereby up-regulating the secretion of chemokines such as SDF-1 and PDGF-BB, which promotes the recruitment of BMSCs and endothelial cells [11]. Titanium ion release can induce pro-inflammatory cytokine secretion in monocytes [9]. Notably, a few studies have reported the effect of strontium on monocytes. Buache et al. [27] reported that strontium-substituted biphasic calcium phosphate particles inhibited pro-inflammatory cytokine release from monocytes. The findings of our study further demonstrated that strontium suppressed the release of pro-inflammatory cytokine in monocytes. However, the impact of locally released Sr^2+^ from functionalized materials on monocyte subset differentiation remains unexplored.

Monocytes are divided into classical and nonclassical subsets based on the cell surface marker Ly6C [5,6]. It was reported that Ly6C^hi^ monocytes promote neutrophil infiltration and reactive oxygen species production, while Ly6C^lo^ monocytes derived from Ly6C^hi^ subsets are closely associated with apoptotic neutrophil clearance [33]. Our results showed that classical monocytes around implants were enriched in pro-inflammatory-cytokine- and osteoclast-related genes, whereas nonclassical monocytes exhibited immunosuppressive properties. A recent study pointed out that a TiO_2_ nanostructured implant regulated the M2c polarization and cytokine secretion profile of inflammatory monocytes by cytoskeleton rearrangement, which was the first metallic implantable material study focusing on the functions of specific monocyte subsets in material-mediated host immune response [34]. Currently, there has been no research investigating the properties of biomaterials affecting the differentiation and function of classical/nonclassical monocytes. Our study demonstrated that Sr-SLA implants suppressed the excessive inflammatory phenotype by reprogramming monocyte subsets. Comparative analysis further revealed that Sr-SLA implants markedly reduced the proportion of classical monocytes while expanding nonclassical subsets. Given the localized Sr^2+^ release from Sr-SLA implants in vivo, we propose that Sr-SLA implants modulate monocyte heterogeneity via Sr^2+^, suppressing early excessive inflammation and establishing an anti-inflammatory, pro-osteogenic immune microenvironment.

Activation of the NLRP3 inflammasome is a critical step in inflammatory responses, driving caspase-1 maturation and subsequent release of the pro-inflammatory cytokines IL-1β and IL-18, thereby exacerbating local inflammation [25]. Tsukalov et al. [35] demonstrated that monocyte activation status and functional properties are responsive to NLRP3 inflammasomes. Seyedsadr et al. [36] found that IL-11 induced NLRP3 inflammasome activation in monocytes, promoting inflammatory monocyte expansion. In this study, combined with the analysis results of scRNA-seq, we also hypothesized that Sr-SLA would down-regulate NOD-like receptor signaling pathways in monocytes. Both in vivo and in vitro results demonstrated that Sr-SLA treatment led to a reduction in the expression of NLRP3-related genes and proteins in monocytes. Further, there were fewer CD11b^+^Ly6C^+^NLRP3^+^ cells observed around Sr-SLA implants compared to those around SLA controls. Additionally, nonclassical monocytes exhibited lower inflammasome-related gene expression than classical subsets. These results indicated that Sr-SLA implants suppressed NLRP3 inflammasome activation in a subset-dependent manner.

Calcium ions, as critical extracellular and intracellular messengers, regulate diverse biological functions through ion channels and transporters [37,38]. Strontium has been shown to promote osteoblast proliferation and differentiation by activating the calcium-sensing receptor (CaSR) and downstream ERK1/2–MAPK pathways while inducing osteoclast apoptosis via CaSR-PKCβII/NF-κB signaling [39,40]. However, the mechanism by which Sr^2+^ regulates monocyte function and osteoimmune microenvironments through calcium channels remains unexplored. TRPM2, an oxidative-stress-sensitive nonselective calcium channel, is predominantly studied for its role in innate immunity and inflammation across disease models. For instance, TRPM2 expression correlates with monocyte-derived CXCL2 secretion and neutrophil infiltration in colitis [26], while TRPM2-deficient mice show reduced IL-12, interferon levels, and nitric oxide synthase expression in splenic macrophages during *Listeria* infection [41]. These findings highlight TRPM2 as a potential target for anti-inflammatory strategies [42]. However, there are few reports on the effect of metal elements on TRPM2.

Our data collectively delineated a sequential signaling cascade through which Sr^2+^ modulates monocyte activation. We found that classical monocytes around implants exhibited up-regulated TRP channel pathways, with TRPM2 showing the highest expression. Both in vivo and in vitro experiments demonstrated that Sr-SLA treatment considerably suppressed TRPM2 expression and its channel activity, as evidenced by reduced intracellular Ca^2+^ accumulation. The pivotal question of whether the observed NLRP3 inflammasome inhibition is a direct consequence of TRPM2 suppression is strongly addressed by our pharmacological intervention. The fact that the TRPM2 inhibitor DPQ phenocopied the effects of SrCl_2_, effectively reversing DOTAP-induced TRPM2/NLRP3 activation, cytokine secretion, and Ca^2+^ fluctuations, strongly indicated that TRPM2 inhibition was the upstream event in this pathway. This is consistent with the established role of TRPM2-mediated Ca^2+^ influx as a key signal for NLRP3 inflammasome assembly [43]. Another study reported that the TRPM2–Ca^2+^–NLRP3 axis has been implicated in lupus nephritis and periodontitis-associated bone defects as a common regulatory node for inflammatory–osteogenic imbalance [44,45]. By linking this axis to biomaterial-mediated immune microenvironments, our study identified TRPM2 as a key molecular target for strontium-doped biomaterial immunomodulation, establishing a link between strontium ions and the TRPM2–Ca^2+^–NLRP3 axis in monocytes. Although the use of DPQ strongly supports the pivotal role of TRPM2, utilizing TRPM2 knockout or knockdown models will be a critical direction for our future research to unequivocally confirm this mechanism. Finally, we demonstrated that conditioned media from Sr-SLA-treated monocytes substantially enhanced BMSC osteogenic differentiation, indicating that Sr^2+^ reshapes the cytokine profile to establish a pro-regenerative microenvironment by regulating the proportion of monocyte subsets. This finding extends the immunological implications of dual “pro-osteogenic/anti-osteoclastic” effects in strontium.

While our study demonstrated that Sr-SLA implants orchestrate a pro-regenerative immune microenvironment during the critical early healing phase, the long-term clinical performance of such functionalized implants is a paramount consideration. Our data confirmed sustained release of Sr ions over 21 d, a period that covered the early inflammatory and initial bone formation stages. In our previous work, the Sr ion release profile of Sr-SLA implants was observed to plateau by day 28 [18], indicating that its release is primarily concentrated in the early postimplantation phase. Furthermore, our results showed no significant increase in serum Sr ion concentration, indicating that the localized release of Sr ions exerts its effects primarily at the implantation site without inducing systemic alterations. From the perspective of long-term biocompatibility, this characteristic of early concentrated release also implies a relatively low risk of long-term cumulative exposure in vivo. Furthermore, it is worth noting that the long-term biocompatibility of Sr-incorporated titanium has been positively indicated in previous studies. Our team has reported that Sr-SLA implants exhibited rapid osseointegration and superior bone parameters (BV/TV%, BIC%) at 4 and 8 weeks [46–48]. In a study by Jiang et al. [48], H&E staining of major organs revealed no apparent pathological changes at 8 weeks postsurgery and rat body weight increased normally throughout the study period. Additionally, other studies also reported superior bone-bonding strength in Sr-loaded implants compared to that in untreated titanium implants at 4, 8, and 12 weeks [49,50]. In the study by Honda et al. [50], 1-year histological observations confirmed that the Sr-loaded implant maintained a native-like diaphyseal bone structure without failure. These collective findings substantiate that Sr-doped titanium-based materials possess favorable long-term stability and biocompatibility. Nonetheless, future investigations monitoring Sr ion release profiles, immune cell responses, and bone remodeling over the long term in large, translational animal models will be crucial to fully validate the long-term efficacy and safety of these immunomodulatory implants before clinical application.

In summary, this study elucidated the molecular mechanism by which Sr-functionalized titanium implants suppress early inflammatory responses and promote bone regeneration through the reprogramming of monocyte subsets and the TRPM2–Ca^2+^–NLRP3 axis. Sr-SLA implants reduced the proportion of pro-inflammatory classical monocytes while expanding nonclassical monocytes, thereby establishing a pro-regenerative immune microenvironment by mitigating early excessive inflammation. Sr ions inhibited TRPM2-mediated Ca^2+^ influx, suppressing NLRP3 inflammasome activation and downstream IL-1β/IL-18 release in monocytes. The expansion of nonclassical monocytes treated with Sr-SLA enhanced the osteogenic differentiation of BMSCs. This work not only advances osteoimmunology but also provides a scientific basis for developing “precision immunomodulatory” implants targeting monocyte–TRPM2 interactions, representing a paradigm shift in biomaterial research from passive “biocompatibility” to active “immune programming”. Future studies should explore the translational potential of these findings to advance personalized implants in complex pathological environments.

## Ethical Approval

All experiments were approved by the Laboratory Animal Welfare and Ethics Review Committee of Zhejiang University, Hangzhou, China (ZJU20250052).

## Data Availability

The datasets used during this study will be made available on reasonable request.
