# Supplementary material for: The Strontium Ion Reprograms Monocyte Subsets via TRPM2 Channel Regulation to Enhance Osseointegration
Source: Biomater Res. 2025 Nov 24;29:0286. doi: 10.34133/bmr.0286 (PMC12641163; doi:10.34133/bmr.0286)
Supplement: Supplementary 1 — Figs. S1 to S8 Tables S1 and S2 [file bmr.0286.f1.docx]

**Supplementary Materials**

**Strontium ion reprograms monocyte subsets via TRPM2 channel regulation to enhance osseointegration**

Congrui Zhao^1†^, Antian Xu^1†^, Jingyao Gong^1^, Yangbo Xu^1^, Ping Sun^1*^, Fuming He^1*^

^1^ Stomatology Hospital, School of Stomatology, Zhejiang University School of Medicine, Zhejiang Provincial Clinical Research Center for Oral Diseases, Key Laboratory of Oral Biomedical Research of Zhejiang Province, Cancer Center of Zhejiang University, Engineering Research Center of Oral Biomaterials and Devices of Zhejiang Province, Hangzhou, Zhejiang, 310008, China

* Address correspondence to: nosleep@zju.edu.cn (P. Sun), hfm@zju.edu.cn (F. He)

† These authors contributed equally to this work.

**This file includes:**

**Fig****ure. S1.** Surface characteristics of SLA and Sr-SLA.

**Figure. S2.** Ion release profile of SLA and Sr-SLA.

**Figure. S3.** Detection of strontium ion concentration in mouse serum before implantation and 3, 7, 14 days post-implantation of Sr-SLA.

**Figure. S4.** The functional enrichment of monocytes.

**Figure. S5.** Differential gene expression in monocyte subclusters.

**Figure. S6.** The expression levels of TRP family genes in monocytes.

**Figure. S7.** CCK8 assay showing the cell activity of monocytes under the treatment of SrCl₂, DOTAP and DPQ.

**Figure. S8.** Correlation analysis of TRPM2 and NLRP3 expression levels.

**Table S1.** Primer sequences used for RT-qPCR.

**Table S2.** Numbers and proportions of ten cell types between SLA and Sr-SLA groups.

**Supplementary Figure 1**


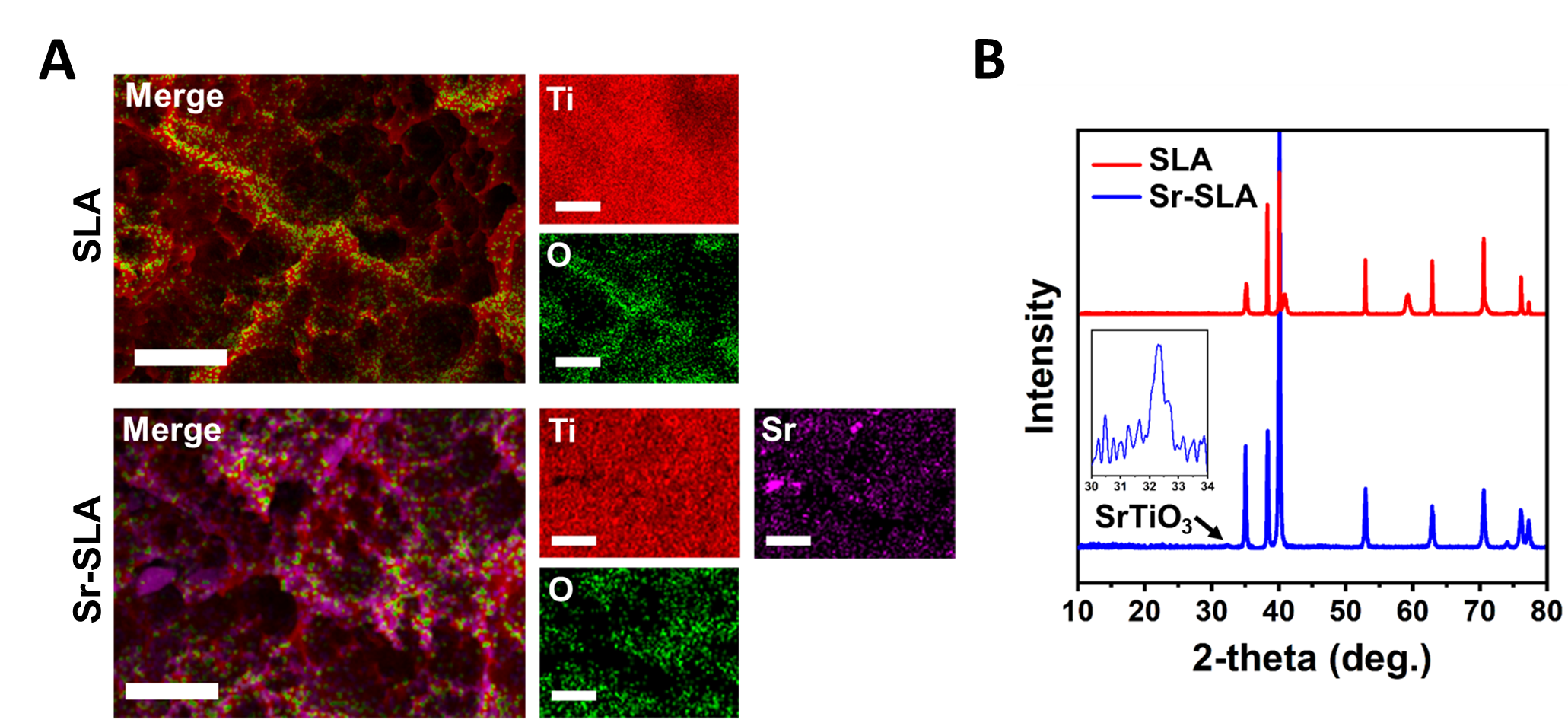


**Figure. S1.** Surface characteristics of SLA and Sr-SLA. (A). EDS mappings of SLA and Sr-SLA surfaces (Scale bar=5μm). (B). XRD patterns of SLA and Sr-SLA surfaces.

**Supplementary Figure 2**

**
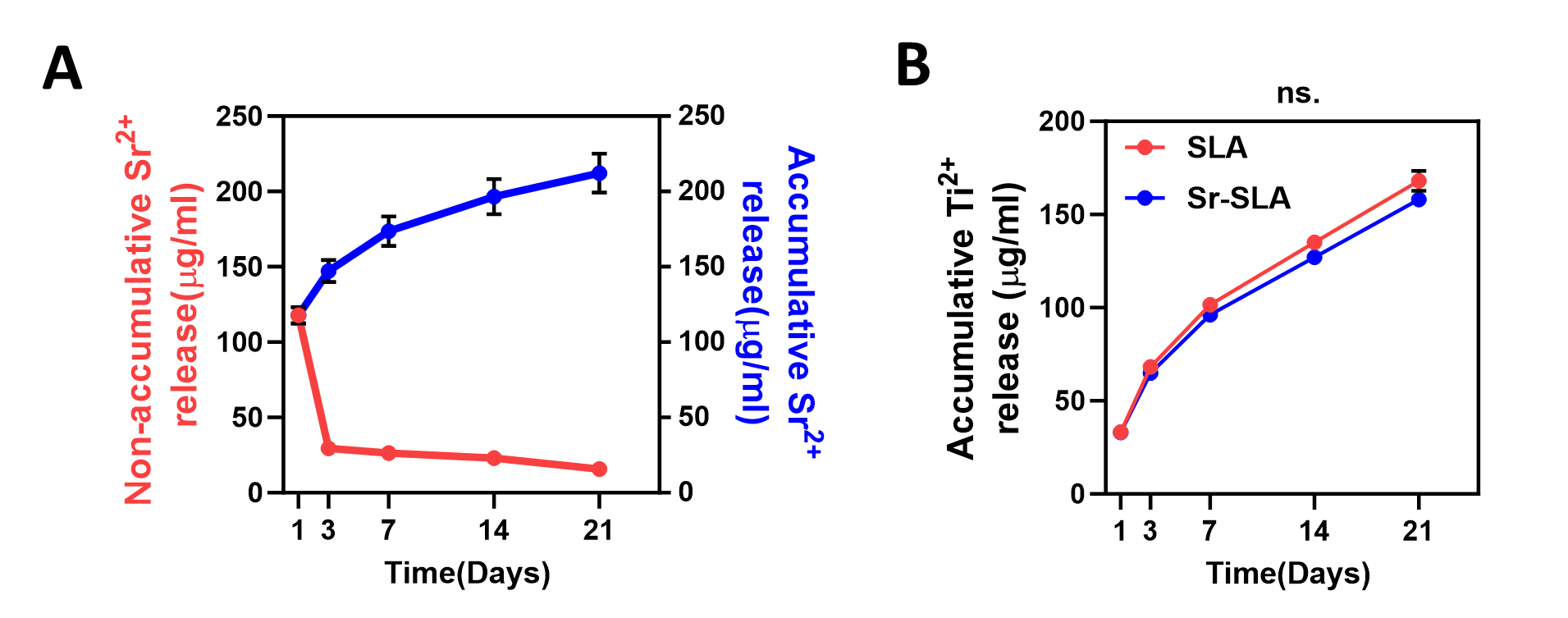
**

**Figure. S2.** Ion release profile of SLA and Sr-SLA. (A). Ion release profile of Sr^2+^ ions from Sr-SLA implants after incubation for 1, 3, 7, 14, 21 days. (B). Ion release profile of Ti^2+^ ions from SLA and Sr-SLA implants after incubation for 1, 3, 7, 14, 21 days. n=3 materials per group and per time point.

**Supplementary Figure 3**

**Figure. S3.** Detection of strontium ion concentration in mouse serum before implantation and 3, 7, 14 days post-implantation of Sr-SLA. n=3 biologically independent samples per time point.

**Supplementary Figure 4**


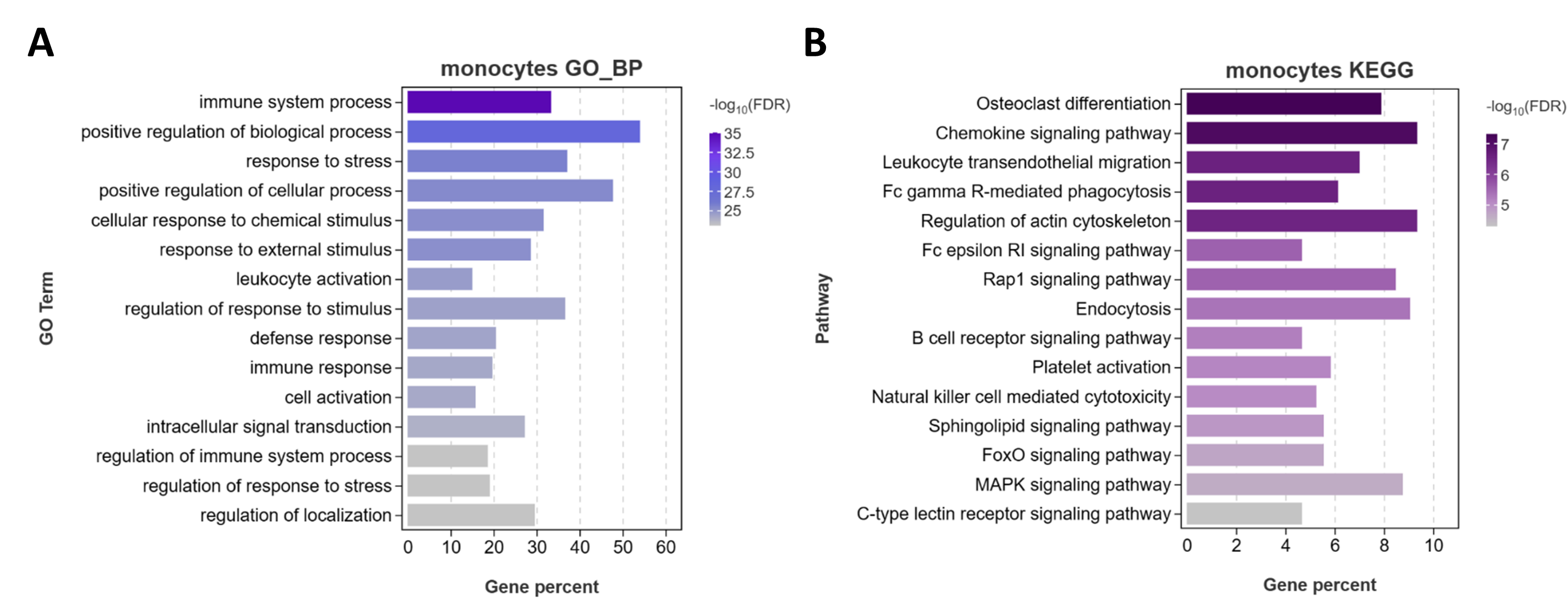


**Figure. S4.** The functional enrichment of monocytes. (A). Barplot showing the GO enrichment of biological function in monocytes. (B). Barplot showing the KEGG enrichment in monocytes.

**Supplementary Figure 5**


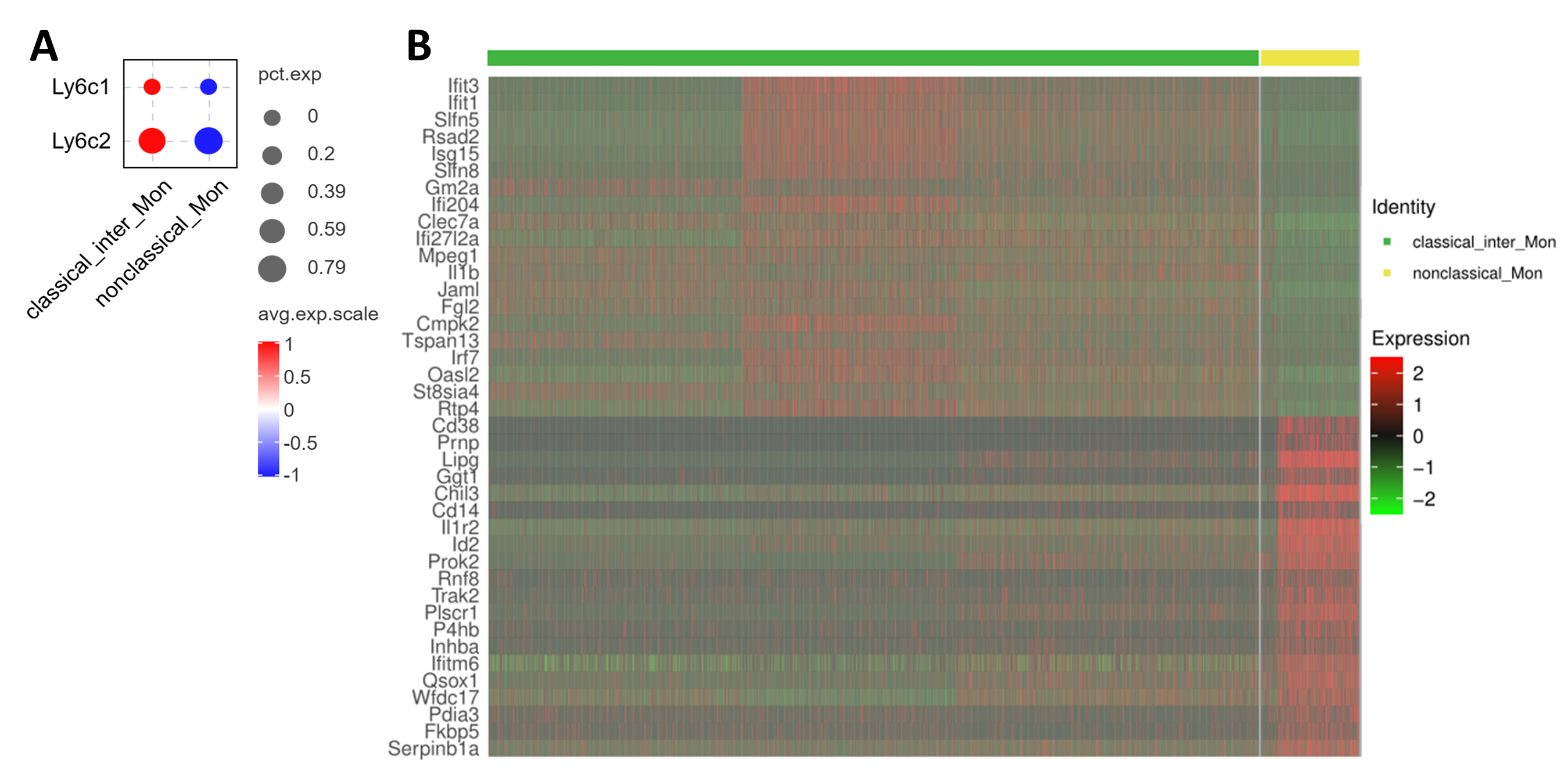


**Figure. S5.** Differential gene expression in monocyte subclusters. (A). Dot plot showing the expression level of Ly6C for monocyte subclusters. (B). Heatmap showing the up-regulate genes for monocyte subclusters.

**Supplementary Figure 6**


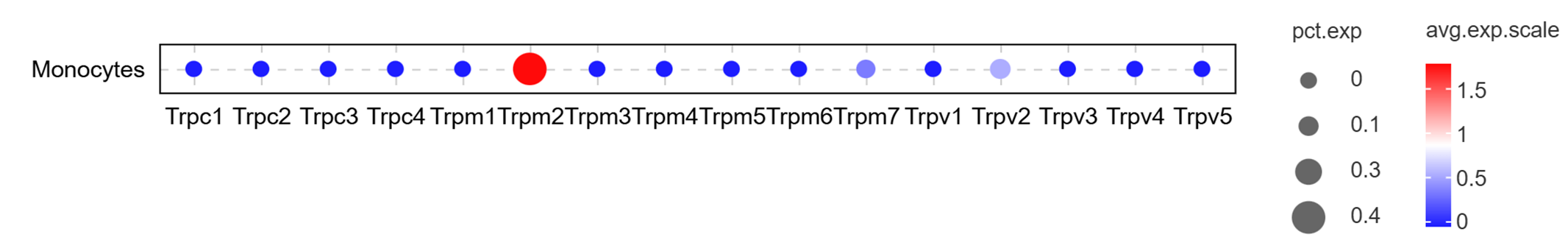


**Figure. S6.** The expression levels of TRP family genes in monocytes.

**Supplementary Figure 7**


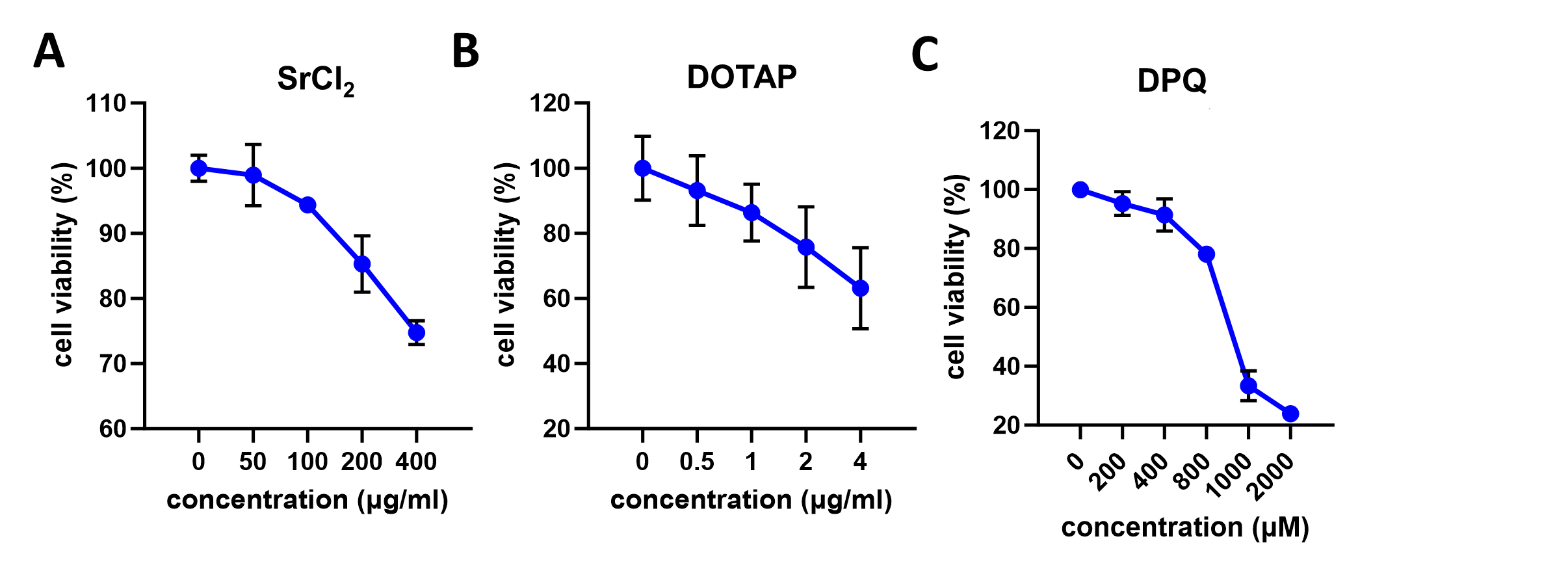


**Figure. S7.** CCK8 assay showing the cell activity of monocytes under the treatment of SrCl₂ (A), DOTAP (B) and DPQ (C). n=3 biologically independent samples.

**Supplementary Figure 8**

**Figure. S8.** Correlation analysis of TRPM2 and NLRP3 expression levels.

**Table S1.** Primer sequences used for RT-qPCR.

| **Gene** | **Direction** | **Primer sequences (5’ to 3’)** |
| --- | --- | --- |
| *Gapdh* | Forward | GGCAAATTCAACGGCACAGTCAAG |
|  | Reverse | TCGCTCCTGGAAGATGGTGATGG |
| *Trpm2* | Forward | CTCTCGGACGCAGGGAAGGTAG |
|  | Reverse | CTGGAAGGTGTGTGGCTTGATGG |
| *Nlrp3* | Forward | GCTGCGATCAACAGGCGAGAC |
|  | Reverse | CCATCCACTCTTCTTCAAGGCTGTC |
| *Casp1* | Forward | ATACAACCACTCGTACACGTCTTG |
|  | Reverse | CAGATCCTCCAGCAGCAACTTC |
| *Asc* | Forward | GAAGTGGACGGAGTGCTGGATG |
|  | Reverse | ATCTTGTCTTGGCTGGTGGTCTC |
| *Il1b* | Forward | CACTACAGGCTCCGAGATGAACAAC |
|  | Reverse | TGTCGTTGCTTGGTTCTCCTTGTAC |
| *Il18* | Forward | GAGACCTGGAATCAGACAACTTTGG |
|  | Reverse | CTGGGGTTCACTGGCACTTTG |
| *Runx2* | Forward | GCAGCAGCAGCAGCAGGAG |
|  | Reverse | GCACGGAGCACAGGAAGTTGG |
| *Bmp2* | Forward | AAGCGTCAAGCCAAACACAAACAG |
|  | Reverse | GAGGTGCCACGATCCAGTCATTC |
| *Alpl* | Forward | CACGGCGTCCATGAGCAGAAC |
|  | Reverse | CAGGCACAGTGGTCAAGGTTGG |
| *Col1a1* | Forward | GACAGGCGAACAAGGTGACAGAG |
|  | Reverse | CAGGAGAACCAGGAGAACCAGGAG |

**Table S2.** Numbers and proportions of ten cell types between SLA and Sr-SLA groups.

| **Cell annotation** | **Number (Proportion (%))** | | |
| --- | --- | --- | --- |
|  | **SLA** | **Sr-SLA** | **Total** |
| Monocyte | 1976 (37.37%) | 3466 (36.22%) | 5442 (36.63%) |
| Neutrophil | 1212 (22.92%) | 2212 (23.12%) | 3424 (23.05%) |
| Macrophage | 627 (11.86%) | 1328 (13.88%) | 1955 (13.16%) |
| B cell | 1026 (19.4%) | 1749 (18.28%) | 2775 (18.68%) |
| T cell | 118 (2.23%) | 200 (2.09%) | 318 (2.14%) |
| Dendritic cell | 107 (2.02%) | 210 (2.19%) | 317 (2.13%) |
| Erythroid cell | 102 (1.93%) | 170 (1.78%) | 272 (1.83%) |
| Basophil | 45 (0.85%) | 108 (1.13%) | 153 (1.03%) |
| Natural killer cell | 34 (0.64%) | 62 (0.65%) | 96 (0.65%) |
| Hematopoietic stem progenitor cell | 41 (0.78%) | 63 (0.66%) | 104 (0.70%) |
